# Validation of the Chinese version of the ‘caring ability of family caregivers of patients with cancer scale (CAFCPCS)’ in family caregivers of elderly patients with cancer: A study protocol

**DOI:** 10.1002/nop2.2227

**Published:** 2024-07-14

**Authors:** Dongmei Zhuang, Yan Wang, Qin Chen, Ting Wang, Peng Zhou, Furong Zhu, Shaohua Hu

**Affiliations:** ^1^ School of Nursing Anhui Medical University Hefei Anhui China; ^2^ Suzhou Hospital of Anhui Medical University Suzhou Anhui China; ^3^ Department of Gastrointestinal Surgery The First Affiliated Hospital of Anhui Medical University Hefei Anhui China; ^4^ Department of Nursing The First Affiliated Hospital of Anhui Medical University Hefei Anhui China

**Keywords:** caring ability, elderly patients with cancer, family caregivers, study protocol, validation

## Abstract

**Aim:**

This study aims to translate the English version of the ‘caring ability of family caregivers of patients with cancer scale (CAFCPCS)’ into Chinese and validate its psychometric properties in the family caregivers of elderly patients with cancer.

**Design:**

A methodological study.

**Methods:**

Based on the Brislin translation model, the original scale will be translated and back‐translated, the Delphi expert consultation method will be adopted for cross‐cultural adaptation, and the pilot will be carried out in 20–30 family caregivers of elderly patients with cancer. Then, a dual‐centre prospective study will be conducted by recruiting 371–542 family caregivers of elderly patients with cancer to validate the psychometric properties of the Chinese version of CAFCPCS.

**Results:**

The scale's content validity will be evaluated using the Delphi expert inquiry method, and the face validity will be evaluated using a pre‐experiment. Exploratory factor analysis (EFA) and confirmatory factor analysis (CFA) will be used to assess structural validity, while internal consistency reliability and split‐half reliability will be used to assess reliability.

**Patient or Public Contribution:**

Public involvement is of great significance for this study. Participants will be used in a pre‐test to give feedback on whether the contents of the clinical pilot version of CAFCPCS after expert consultation can reflect real problems and whether the sentences can be well understood. Based on their opinions, the research group will further refine the scale.

## 1 INTRODUCTION

Global Cancer Statistics 2020 points out that cancer is the leading cause of death in countries around the world and an important obstacle to life span (Sung et al., 2021). In 2020, the estimated new cancer cases and deaths worldwide were 19.3 million and nearly 10 million, respectively (Cao et al., 2021). China was ranked top in the world with 4.57 million new cases and 3 million deaths (WHO, 2020). Since 2000, the number of cancer cases, deaths, crude incidence rate, and mortality rate in China have gradually increased (Xia et al., 2022). Age is the most common risk factor for cancer incidence and the development of malignant tumours (Jackaman et al., 2017). In China, the incidence rate of malignant tumours has increased significantly after the age of 40 and reached the highest among the age group of 75–80. The trend of mortality with age recapitulates that of incidence rate (Cao & Chen, 2019). It is expected that by 2050, the population of China aged 65 and above will reach 400 million, accounting for 26.9% of the total population (Fang et al., 2015).

Moreover, in elderly patients with cancer, their declining physical functioning (Akechi et al., 2012; Derks et al., 2016; Nightingale et al., 2021) results in higher hospitalisation costs, length of stay, complications, and mortality rates than the rest of the population (Gbeasor‐Komlanvi et al., 2020). Furthermore, their preferences, needs, and values are also different from other populations, so caring for them is complex (Akechi et al., 2012). This is extremely challenging for both healthcare providers and caregivers.

With medical advancement, the life span of patients with cancer has gradually extended, and their main care environment has shifted from hospitals to households. So, family caregivers play a major role in care tasks (Ferrell et al., 2013). However, providing high‐quality care requires family caregivers to possess sufficient knowledge and skills, as well as awareness of relevant patient information and their illnesses (Given et al., 2008; Hellesø et al., 2012). Therefore, it is crucial to evaluate whether a family caregiver has the caring ability to meet the care needs of patients. But the fact is that family caregivers do not have the preparation and awareness to play their important role, nor do they receive more support (Kristanti et al., 2018).

Therefore, in this case, the professional caregiver should empower the family caregiver and help them acquire the ability to meet the patient's care needs. The conceptual framework of empowerment theory includes professional nursing staff working together with family caregivers to develop care plans based on their existing problems, enabling them to acquire care knowledge, skills, and resources, achieve problem‐solving, improve care capabilities, manage their own lives, and improve their quality of life (Park et al., 2015). Multiple studies have confirmed that this approach can improve the caregiving ability of family caregivers (Iswanti et al., 2024; Mardhiyah et al., 2022; Zhao et al., 2021).

It is not difficult to see that it is very meaningful to evaluate the caring ability of family caregivers of elderly patients with cancer. On the one hand, the ability of family caregivers to take care of patients has made a significant impact on their general care, thereby affecting their recovery rate and adaptability to cancer (Nemati et al., 2020); On the other hand, it helps to understand the strengths and weaknesses of family caregivers as well as helps healthcare professionals more accurately develop intervention measures to empower them to provide high‐quality care (Sakanashi & Fujita, 2017).

## 2 BACKGROUND

Many researchers have developed tools to evaluate the caregiving ability of family caregivers, including “Caregiver Task Inventory (CTI),” “Modified Version of Caregiver Task Inventory (MV‐CTI),” and “Caregiver Reaction Assessment (CRA).” But most of the applications are for family caregivers of elderly patients (Clark & Rakowski, 1983), elderly patients with stroke (Lee & Mok, 2011), and patients with Alzheimer's disease (Given et al., 1992), without considering the particularity of cancer. Patients with cancer have serious physical and mental problems, requiring a large amount of healthcare resources, and family support (Bajwah et al., 2020). The National Alliance for Caregiving (NAC) study also shows that there are approximately 3.18 million caregivers of patients with cancer in the United States, and cancer ranks fifth among the main issues or diseases that require caregiver care (NAC, 2020).

Based on the above reasons, it is crucial to choose appropriate tools to evaluate the care ability of family caregivers for elderly patients with cancer.

In 2020, Nemati and Rassouli et al. used a sequential exploratory mixed‐method to design the ‘caring ability of family caregivers of patients with cancer scale (CAFCPCS)’, which resulted from qualitative and quantitative stages. The scale has been proven to be an effective and reliable tool and has been validated in “mothers of children with cancer” (Khademi et al., 2022; Nemati et al., 2020). The CAFCPCS has 5 dimensions and 31 items. The overall scale's and all dimensions' internal consistency coefficients (Cronbach's a) are 0.934, 0.705–0.933, respectively; The intraclass correlation coefficient (ICC) of the total scale and all dimensions are 0.944, 0.734–0.932. The positive dimensions (effective role play and trust) scores are: 1 = Strongly disagree, 2 = Disagree, 3 = No idea, 4 = Agree, and 5 = Strongly agree; The negative dimensions (fatigue and surrender, uncertainty, and caring ignorance) scores are opposite to the positive dimensions.

Thus far, a validated Chinese version of CAFCPCS is not yet available. Therefore, the purpose of this study is to translate CAFCPCS into Chinese and test its psychometric properties through reliability and validity among the family caregivers of elderly patients with cancer.

## 3 THE STUDY

### 3.1 Aims

Translation and evaluation of the psychometric properties of the CAFCPCS for Chinese family caregivers of elderly patients with cancer.

### 3.2 Design

This is a methodological study that will involve two medical centres.

### 3.3 Methods

#### 3.3.1 Study setting and timeline

The location will be two districts of the First Affiliated Hospital of Anhui Medical University in Hefei, Anhui Province, and two districts of Suzhou Hospital of Anhui Medical University in Suzhou, Anhui Province.

The entire investigation is anticipated to last 18 months, from October 1, 2022, to March 31, 2024.

### 3.4 Participants and recruitment

Family caregivers with clinically confirmed elderly patients with cancer will be recruited from the above two medical centres.

#### 3.4.1 Sample size

We will collect two independent samples, one for exploratory factor analysis (EFA) and one for confirmatory factor analysis (CFA), using the convenience sampling method.

#### 3.4.2 Sample 1

The rule of thumb for EFA is to recruit 5–10 samples for each variable and an additional 10% of samples to avoid potential losses, which means that sample 1 will require 171–342 participants (Garrote‐Cámara et al., 2022; Winnige et al., 2020).

#### 3.4.3 Sample 2

Following the rule of thumb for CFA, sample 2 shall not be less than 200 samples (Huang et al., 2022).

Therefore, the sample size required for this study will be 371–542.

##### Inclusion criteria

(1) Reported as a family caregiver for an elderly patient with cancer (age 60 years or above). (2) Aged 18 and above responsible for the patient's primary care tasks. (3) Informed consent and voluntary participation.

##### Exclusion criteria

(1) Unable to communicate in Mandarin. (2) Receiving compensation (such as nannies, nursing workers, etc.).

##### Sample characteristics

The participant characteristic questionnaire was designed by the research team based on the literature review and clinical experience. This includes two parts: patient information and family caregiver information (Table 1).

**TABLE 1:** The participant characteristic questionnaire.

Part 1: Patient information
Age
Gender
Education level
Marital status
Cancer species
Other chronic diseases
Payment method for medical expenses

Part 2: Family caregiver information
Relationship with patients
Age
Gender
Education level
Marital status
Work status
Disease or not
Income
Other caregivers except you
Live with the patient
Average daily time spent caring for patients
The time taken care of the patient (after illness)
Previous experience in caring for patients
Place of residence

The first author of this study will receive the written informed consent form. Anytime, for any reason, subjects are free to leave the study without any repercussions.

### 3.5 Data collection methods

Obtain approval from the relevant departments of the selected hospital. The research personnel will distribute informed consent forms and questionnaires, introduce the research purpose, questionnaire filling methods, etc., using unified guidance language and one‐on‐one filling. Family caregivers complete the form, and research personnel promptly retrieve it, check the completeness of the questionnaire, as well verify any incomplete or unclear information.

### 3.6 Data management

The data collected will not be sent in any way and will be stored on a computer that requires a password. Only participating researchers have access.

### 3.7 Study procedures

The Brislin translation model will be used in this study (Brislin, 1970).

#### 3.7.1 Phase 1

Translate the developed English CAFCPCS into Chinese.

##### Stage 1

Two separate translators will complete the forward translation of the original scale. Two translators are bilingual native Chinese speakers who are proficient in English, with different major backgrounds (1 postdoctoral fellow in oncology and 1 senior professional title English teacher), and will compile a written report for each translation (A and B). The research group, which includes 1 nursing graduate supervisor (Ph.D., professor) and 6 nursing graduate students, will then compare them in order to identify any phrasing discrepancies or ambiguities in the language before integrating translation AB.

##### Stage 2

Translate AB backward into English by two bilingual native English speakers who are completely unaware of the original text. In addition, in order to minimize the bias, it is required that the two translators have no medical background knowledge. Translators will write reports for all backward translation versions (C and D), which will then be compared by the research group before formulating the first Chinese version (CD), to check for ambiguous language, errors, etc.

##### Stage 3

The Delphi consulting experts will review the first Chinese version (CD) and develop a clinical pilot version of CAFCPCS. The expert group will be comprised of 15 members and based on the following criteria: (1) working in cancer‐related areas, (2) having at least 10 years of clinical work experience, (3) having intermediate or above professional titles, and (4) having bachelor's degree or above. The requirement is that after each round of expert consultation, the research team will summarise and analyse the expert opinions, and modify each item according to the expert opinions. On this basis, the next round of consultation questionnaire will be prepared, indicating the adjustment of the items and consulting the experts again. Repeat the above process until expert opinions converge and stop consulting. Moreover, to ensure that experts complete the consultation questionnaire within the specified time, they will be reminded of the timeline 2 days before the questionnaire is collected, with an interval of 2–3 weeks between each round of consultations.

**FIGURE 1:**
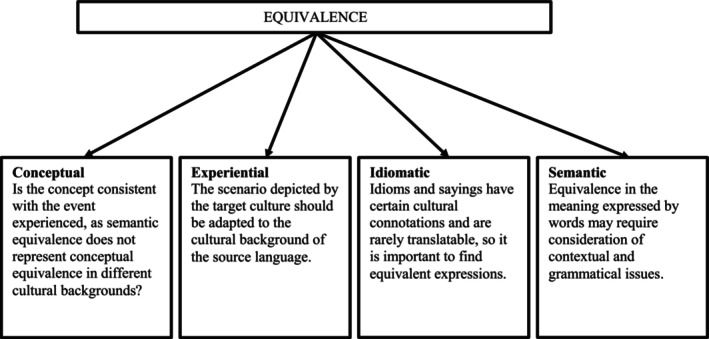
Cross‐cultural equivalence areas between original and Chinese versions.

The above process follows the cross‐cultural guidelines and aims to achieve equivalence between the original text and Chinese, including conceptual, experiential, idiomatic, and semantic equivalence, as shown in Figure 1 (Guillemin et al., 1993).

##### Stage 4

Pretesting will be conducted over 20–30 family caregivers of elderly patients with cancer with the goal of assessing whether the clinical pilot version of CAFCPCS is clear, concise, and easy to understand. Based on this, a clinical application version will be formed.

#### 3.7.2 Phase 2

Validate the psychometric properties of the Chinese version of the CAFCPCS.

**FIGURE 2:**
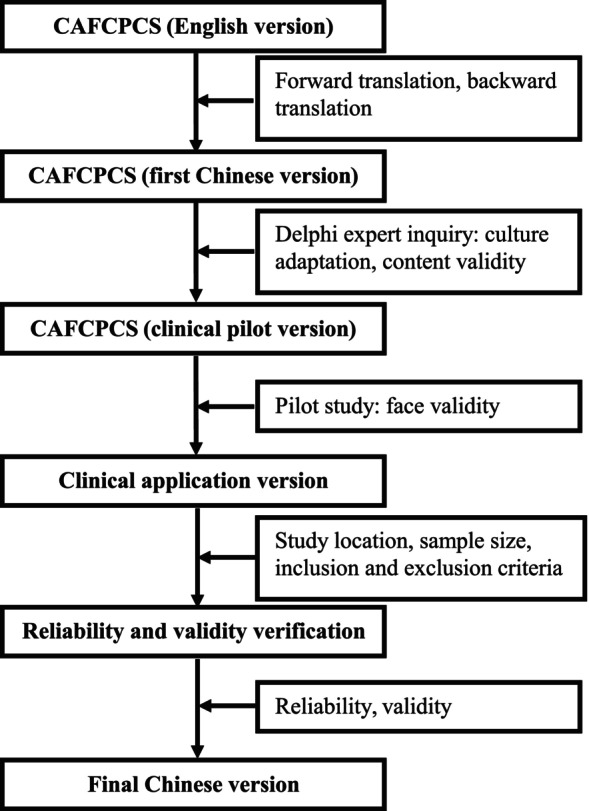
Flowchart of the study protocol of the Chinese version of the CAFCPCS.

Two family caregiver samples of elderly patients with cancer will be employed for psychometric validation on the clinical application version of CAFCPCS and to determine the final Chinese version. A flowchart of the study protocol is shown in Figure 2.

### 3.8 Data analyses

Establish a database using Excel, double entry, and then use IBM SPSS 23.0 and Amos 26.0 software to analyse all the data. For quantitative data, mean and standard deviation (SD) or median and interquartile range will be calculated depending on the distribution, while for qualitative data, frequency and percentage will be calculated. T‐test will be used to compare the differences between two sets of data. The statistically significant difference is between *p* < 0.05 or *p* < 0.01.

#### 3.8.1 Item exploration

##### Correlation coefficient analysis

The correlation between the item score and the total score of the scale will be calculated. A correlation coefficient is defined as weak if the value is 0.2–0.39, moderate if 0.4–0.49, and strong if 0.50 or above (Ripamonti et al., 2022). Items with a correlation coefficient <0.2 will be deleted (Adel et al., 2022).

##### Critical ratio method

Arrange the total score of the samples in descending order, and use the independent sample *t*‐test to compare the differences between high (top 27% of the total score) and low (bottom 27% of the total score) groups on each item. Items with a critical ratio >3 (*p* < 0.05) will be considered acceptable (Yang, Zhang, Leng, Fan, & Luo, 2022; Yang, Wang, & Wang, 2022).

#### 3.8.2 Reliability

##### Internal consistency reliability

The internal consistency reliability is represented by Cronbach's a, and ≥0.7 will be considered acceptable (Núñez‐López et al., 2022).

##### Split‐half reliability

The scale is divided into two parts, and the correlation coefficient of the two parts is calculated as the split‐half reliability, and >0.7 will be considered acceptable (Yang, Zhang, Leng, Fan, & Luo, 2022; Yang, Wang, & Wang, 2022).

### 3.9 Validity

#### 3.9.1 Face validity

Used to evaluate participants' reading comprehension of the content expressed in the item and whether the item can truly achieve the test purpose. Completed during the pre‐testing phase.

#### 3.9.2 Content validity

Using expert inquiry method to calculate content validity. Experts rate each item using the Likert 4‐point scoring system (1 = uncorrelated, 2 = weakly correlated, 3 = relatively correlated, 4 = strongly correlated). In addition, for each domain, we require participants to provide modifications and comments on the items in the free description section. Divide the number of people with scores of 3 and 4 by the total number of people to calculate the item‐level content validity index (I‐CVI). Based on the average, we will calculate the scale‐level content validity index (S‐CVI/Ave). An I‐CVI value of 0.78 or above and an S‐CVI/Ave value of 0.90 or above are acceptable standards (Sekine et al., 2022).

### 3.10 Construct validity

#### 3.10.1 (1) Exploratory factor analysis

EFA is used to determine the potential structure of the Chinese version of CAFCPCS. First of all, it demands to perform the Kaiser–Meyer–Olkin (KMO) measure of sampling adequacy and Bartlett's Test of Sphericity. When the KMO value is greater than 0.7 and Bartlett's Test of Sphericity is statistically significant (*p* < 0.05), it indicates that factor analysis is suitable (Pires et al., 2022). In the next place, it needs to determine the number of common factors, using principal component analysis and maximum variance orthogonal rotation method to extract common factors with feature values >1. Whether items will be retained depends on their factor load. Items will be deleted if their factor loadings are <0.3; between 0.3 and 0.6, acceptable; above 0.6, good (Khanjari et al., 2022).

#### 3.10.2 (2) Confirmatory factor analysis

CFA is used to verify whether the corresponding relationship between factors obtained from EFA and scale items is consistent using the maximum likelihood method. The goodness of fit depends on the chi‐square degree of freedom ratio (χ^2^/df), comparative fit index (CFI), Tucker Lewis index (TLI), approximate root mean square error (RMSEA), and standardized root mean square residual (SRMR). If χ^2^/df <5, acceptable; <3, desirable. CFI and TLI >0.90, acceptable; >0.95, good. RMSEA and SRMR <0.08, acceptable; <0.05, ideal (Bautista et al., 2018; Teoman & Harmancı Seren, 2022).

## 4 DISCUSSION

Although the increased aging population reflects longevity, it also increases the diseases opportunities including cancer, which is currently the leading cause of death worldwide (Lozano et al., 2012; Silva et al., 2022). With the progress of cancer treatment, the survival rate of elderly patients with cancer is gradually improving (Galvin et al., 2022). Family members play an important role in cancer care management (Sun et al., 2021). However, the caring ability of family caregivers varies by the patient's care needs. Therefore, it is important to develop an evaluation tool to assess whether their caring ability can meet the patient's care needs. The purpose of this study is to fill this gap. Using this tool will not only help professional caregivers understand the strengths and weaknesses of family caregivers in order to develop better plans to improve their nursing abilities but also assess whether the nursing plans provided by professional caregivers are sufficient and effective.

### 4.1 Strengths and limitations

This research protocol outlines the translation and validation process of the Chinese version of the ‘caring ability of family caregivers of patients with cancer scale (CAFCPCS)’, which was approved by the scale developer and will follow strict cross‐cultural guidelines.

Employing elderly cancer patient family caregivers and experts‐led process in phase 1, 15 experts will conduct Delphi consultation and 20–30 elderly cancer patient family caregivers will conduct a pilot study, in order to establish face and content validity as well as cross‐cultural adaption for CAFCPCS in China.

In phase 2, two bigger samples of family carers for elderly patients with cancer will each be used to validate the psychometric properties of the Chinese CAFCPCS.

However, this study will only include family caregivers of elderly patients with cancer, which may have selection bias. In future studies, different study populations can be included to further validate the effectiveness of the scale.

## 5 CONCLUSIONS

The rigorously designed Chinese version of CAFCPCS will become an effective and reliable tool for medical institutions to evaluate the nursing ability of home caregivers for elderly patients with cancer, providing a basis for professional nursing staff to develop nursing measures to improve the nursing ability of home caregivers.

## AUTHOR CONTRIBUTIONS

DZ designed the study protocol and drafted the manuscript. SH and QC made revisions and related suggestions to the study protocol. TW, PZ, YW, and FZ made a significant contribution to the writing of the manuscript. All authors rigorously reviewed the manuscript and approved publication of this edition.

## FUNDING INFORMATION

This work was supported by the 2023 Graduate Seedling Cultivation Project of School of Nursing, Anhui Medical University (Grant No. hlqm12023016). The 2021 Provincial Quality Engineering Project of Anhui Higher Education Institutions (Grant No. 2021jyxm0718) and the 2021 Natural Science Research Project of Anhui Higher Education Institutions (Grant No. KJ2021ZD0020).

## CONFLICT OF INTEREST STATEMENT

The authors declare no conflict of interest.

## RESEARCH ETHICS COMMITTEE APPROVAL

Ethical approval was received from the Biomedical Ethics Committee of Anhui Medical University (84230018). The results will be shared with participants, Cancer Care Societies, clinical healthcare professionals, and researchers through peer‐reviewed journals and conferences.

## STATEMENT

This study may provide nurses with a tool to measure the caregiving ability of family caregivers of elderly patients with cancer. Through the measurement, nurses can know the caring ability of family caregivers, and provide a basis for formulating targeted measures to improve the caring ability of family caregivers. Therefore, this study is closely related to nurses and nursing.

## Data Availability

The authors will supply the data on request.
